# Brain Activity and Functional Connectivity Patterns Associated With Fast and Slow Motor Sequence Learning in Late Middle Adulthood

**DOI:** 10.3389/fnagi.2021.778201

**Published:** 2022-01-13

**Authors:** Maite Aznárez-Sanado, Luis Eudave, Martín Martínez, Elkin O. Luis, Federico Villagra, Francis R. Loayza, María A. Fernández-Seara, María A. Pastor

**Affiliations:** ^1^School of Education and Psychology, University of Navarra, Pamplona, Spain; ^2^Neuroimaging Laboratory, Center for Applied Medical Research (CIMA), University of Navarra, Pamplona, Spain; ^3^Institute of Biological, Environmental and Rural Sciences (IBERS), Aberystwyth University, Aberystwyth, United Kingdom; ^4^Faculty of Mechanical Engineering and Production Sciences (FIMCP), Escuela Superior Politecnica del Litoral (ESPOL), Guayaquil, Ecuador; ^5^Instituto de Investigación Sanitaria de Navarra (IdiSNA), Pamplona, Spain; ^6^Department of Radiology, Clínica Universidad de Navarra, Pamplona, Spain; ^7^School of Medicine, University of Navarra, Pamplona, Spain

**Keywords:** motor sequence learning, fMRI, connectivity, aging, fast learning, slow learning

## Abstract

The human brain undergoes structural and functional changes across the lifespan. The study of motor sequence learning in elderly subjects is of particularly interest since previous findings in young adults might not replicate during later stages of adulthood. The present functional magnetic resonance imaging (fMRI) study assessed the performance, brain activity and functional connectivity patterns associated with motor sequence learning in late middle adulthood. For this purpose, a total of 25 subjects were evaluated during early stages of learning [i.e., fast learning (FL)]. A subset of these subjects (*n* = 11) was evaluated after extensive practice of a motor sequence [i.e., slow learning (SL) phase]. As expected, late middle adults improved motor performance from FL to SL. Learning-related brain activity patterns replicated most of the findings reported previously in young subjects except for the lack of hippocampal activity during FL and the involvement of cerebellum during SL. Regarding functional connectivity, precuneus and sensorimotor lobule VI of the cerebellum showed a central role during improvement of novel motor performance. In the sample of subjects evaluated, connectivity between the posterior putamen and parietal and frontal regions was significantly decreased with aging during SL. This age-related connectivity pattern may reflect losses in network efficiency when approaching late adulthood. Altogether, these results may have important applications, for instance, in motor rehabilitation programs.

## Introduction

Understanding the relationship between human motor learning capacity and aging becomes more relevant as the world’s population ages (King et al., 2013). Learning a novel motor sequence can be defined as the process by which a series of simple or complex individual movements begin to be executed jointly and without effort as a consequence of motor practice (Willingham, 1998). For this reason, learning a novel motor sequence implies to memorize the sequence of required movements, monitor motor performance and consciously inhibit actions that are not related to the execution of the novel motor sequence *per se*. As a result, learning efficiency, motor performance and control processes are closely related (Ren et al., 2013).

The acquisition of novel motor skills goes through different phases. Fast learning (FL) phase takes place when the learner initiates the practice of the novel motor task and is usually accompanied by fast improvements in motor performance (e.g., reduction of reaction time, increase of correct movements or greater timing accuracy). During this learning phase, considerable improvement in performance can be achieved within minutes (Classen et al., 1998; Muellbacher et al., 2002; Xiong et al., 2009). Continued practice of the novel motor task depends on consolidation processes, and usually results in incremental gains in performance, until the movement is carried out automatically [slow learning (SL) phase] (Doyon, 2008). This last stage of learning is slow and may require several sessions (or weeks) of practice (Nudo et al., 1996). As training progresses, motor performance becomes fluid and less attention is required to perform the movement.

Although motor sequence learning has been studied extensively, contradictory results are still observed, probably due to the implementation of different experimental designs and motor tasks, which do not allow to have a consensual model of motor learning (Halsband and Lange, 2006). Different models have been proposed to explain the operability of motor sequence learning, such as the hierarchical editor model (Rosenbaum et al., 1984), the Cobalt Theory (Willingham, 1998), the Hikosaka model (Hikosaka et al., 2002), the dual processor model (Abrahamse et al., 2013), the cognitive framework for sequential motor behavior (Verwey et al., 2014), or the Doyon and Benali (2005) and Doyon et al. (2018) model, one of the most cited motor learning models. According to existing literature, neural substrates of motor learning evolve over the course of time. Learning a motor sequence implies dynamic interactions between brain structures for the development of optimized motor representations and, consequently, initial phases of learning require more neural resources than when reaching motor automaticity (Jenkins et al., 1994; Toni et al., 1998; Ungerleider et al., 2002; Penhune and Doyon, 2005; Poldrack et al., 2005; Fernandez-Seara et al., 2009; Xiong et al., 2009; Ma et al., 2010; Reithler et al., 2010; Aznarez-Sanado et al., 2013; Wiestler and Diedrichsen, 2013; Wymbs and Grafton, 2015; Karim et al., 2017). Specifically, Doyon et al. (2018) suggest that different cortico-striatal and cortico-cerebellar pathways and limbic structures (i.e., striatum, cerebellum, prefrontal, parietal cortices, and hippocampus) interact during FL to perform a novel motor routine. As learning consolidates, the demand for brain resources is reduced, and cortical and hippocampal areas are compromised to a lesser extent. Once the subject performs a motor task automatically, the long-term representation of the motor routine is stored mainly in cortico-striatal or cortical-cerebellar networks, depending on whether motor sequence learning or motor adaptation tasks are being carried out.

Functional Magnetic Resonance Imaging (fMRI) studies related to motor sequence learning in young subjects are abundant (Tamás Kincses et al., 2008; Reithler et al., 2010), but this is not the case for the aging population. The study of motor sequence learning in elderly subjects is of particularly interest since it has been demonstrated that structural and functional changes in the brain occur across the lifespan. Therefore, motor sequence learning related neural structures might also be affected by age (King et al., 2013). Specifically, the frontal cortex is one of the most vulnerable regions with aging. Previous studies have reported decreases in gray matter volume and degeneration of white matter tracts in elderly subjects that affect frontal regions and their connectivity with subcortical structures (Allen et al., 2005; Raz et al., 2005). These age-related declines in the frontal lobe have been associated with deficits in attention and executive functions (Zimmerman et al., 2006). In addition, two different mechanisms have been described in aging. On one hand, a dedifferentiation process, where a loss of divergence of cognitive and motor abilities occurs (Ren et al., 2013) and results in a shift to less specific neural networks that subserve different functions (Chen et al., 2011). On the other hand, there are compensatory processes, manifested by a broader recruitment of areas for a specific function, trying to reduce the negative impact of aging on cognitive and motor activities (Heuninckx et al., 2005; Ward, 2006).

Regarding motor learning and aging, the existing literature reports that older adults present alterations in the initial acquisition of movement sequences only under conditions of greater task complexity; poor memory consolidation of learned motor sequences; and alterations in motor adaptation in the exposure phase, but not post-exposure, which could suggest a relative preservation of the adaptive capacity of older adults (King et al., 2013). Findings related to the performance of complex motor sequences have shown structural and functional alterations associated with aging in fronto-striatal networks involved in motor learning (Kennedy and Raz, 2005; Raz et al., 2005; Paquet et al., 2008; Bennett et al., 2011). Other studies have reported greater activation of ventrolateral and medial premotor regions in older adults when compared to young adults during an implicit learning task (McGregor et al., 2009). These changes were attributed to a greater perception of complexity of the motor task in the elderly subjects. Similarly, Turesky et al. (2016) reviewed studies that compared simple novel motor movements between young and elderly adults, reporting that older adults recruited additional brain resources located in the supplementary motor area (SMA) (extending to premotor areas) and in sensorimotor regions. Our previous work also identified regions related to spatial and temporal components of motor sequence learning in healthy aged population (Eudave et al., 2017). Increasing age has also been previously associated with greater activation in regions related to selective attention (i.e., fronto-striatal and temporo-parietal regions) (Rubia et al., 2010).

Most of preceding experiments have focused on learning related changes in brain activity. However, studies based on functional connectivity changes over the course of learning are still relatively scarce. Different authors have explored changes in post-learning resting state functional connectivity, reporting changes in connectivity within the motor system in young subjects (Gregory et al., 2014; Stagg et al., 2014; Sampaio-Baptista et al., 2015), but few works have compared resting state networks between young and older subjects (Mary et al., 2017; Michels et al., 2018). Few studies have indeed assessed changes in functional connectivity during learning. In these cases, it has been found in young subjects that: (1) the gain in learning performance is achieved by dynamically adjusting interregional connectivity within the motor network (Ma et al., 2010); and (2) decreased connectivity between the putamen with other regions (i.e., left inferior frontal gyrus and left middle cingulate cortex) as learning progresses (Karim et al., 2017).

In this experiment, we aimed to study motor sequence learning in late middle adulthood by means of assessing motor performance, brain activity and functional connectivity patterns over the course learning. In addition, we also aimed to evaluate whether these patterns were correlated with age. For this purpose, we carried out a longitudinal study where a subsample of the subjects performed a novel motor sequence during 3 months. The existing literature, with its diversity of experimental protocols, analytical approaches and lack of studies in aging population, does not currently provide a consistent picture of learning-related changes in late middle adulthood.

According to existing literature, we expected that: (1) late middle-aged subjects were able to learn and automate the novel motor task, showing significant improvements in motor performance from FL to SL; (2) greater motor efficiency during SL would be associated with the recruitment of reduced neural resources, (3) brain connectivity patterns would change over the course of learning, and (4) age-related differences in brain activity and functional connectivity would be located mainly in frontal regions.

## Materials and Methods

### Participants

Twenty-five healthy subjects [36% female, mean age = 56.3 years old (*SD*: 8.6, *MIN:* 42, *MAX:* 72), see Supplementary Table 1 for detailed description] participated in the first part of the study (Study 1). A subgroup of these participants [11 subjects, 27.3% female, mean age = 54.6 years old (*SD*: 7.3, *MIN:* 45, *MAX:* 67)] were followed-up for 3 months (Study 2, see Section “Study 2 – Fast and Slow Learning Phases”). All of the subjects were right-handed [mean dexterity scores: Study 1: 47.5 (*SD*: 3.3) out of 50; Study 2: 47.5 (*SD*: 3.6) out of 50] as determined by the 10-item Edinburgh handedness inventory (Oldfield, 1971). Each of the items was scored from 1 to 5 and a high score indicated right-hand performance. The total score was calculated by adding the individual item scores. Cognitive dysfunction was assessed with the Mini Mental State Exam (MMSE) test (Folstein et al., 1975). All subjects scored 27 or above in this test.

Exclusion criteria of the study were those associated with Magnetic Resonance Imaging (MRI) use, the presence of cognitive dysfunction or left-handedness. The study was approved by the Committee for Ethics in Research of the University of Navarra. All subjects signed an informed consent prior to joining the study.

### Experimental Setup

Subjects were placed inside the scanner. Task instructions were then projected onto a screen that was visualized by the participants. Motor finger responses from the right hand were collected via a 4-key response pad (Current Designs, Inc.) placed on the abdomen of the participants. Cogent (Cogent 2000, UCL, London) and Matlab were used for task presentation and data collection.

### Experimental Task

#### Study 1 – Fast Learning Phase

Motor sequences were visually presented on a screen to the participants with colored circles, indicative of eight targets of finger movements. Once the sequence of eight targets had been shown, a fixation cross appeared to announce the onset of the sequence reproduction period, which was 12.5 s (s) long. The subjects were requested, during this fixation cross period, to reproduce the same sequence of eight sequential finger movements with their right hand, by pressing the 4 buttons on the response pad [which corresponded to index (1), middle (2), ring (3), and little (4) fingers, respectively]. Subjects were required to reproduce the motor sequence immediately after visualization at the same frequency as it was presented. Two different motor sequences were presented to the subjects, novel and control. The novel sequence to be learned was 2–3–1–3–4–2–1–4. The control sequence consisted of successive finger movements (4–3–2-1–4–3–2–1). Subjects practiced the control but not the novel sequence before entering the scanner. Both tasks (novel and control) were presented in an event related design in blocks of two. At the end of control trials, a 4 s rest condition was added, where subjects were asked to relax and look at the fixation cross. Visual stimuli were presented sequentially on the screen at a frequency of 0.67 Hz [interstimulus interval of 1,500 ms (ms)] and a random jitter was included (0–500 ms) at the beginning of the presentation of each motor sequence. Novel and control trials were repeated 16 times. Subjects underwent one scanning session. No feedback on performance was provided to subjects.

#### Study 2 – Fast and Slow Learning Phases

After the first fMRI session, a subgroup of subjects (*n* = 11, see Section “Participants” for characteristics of participants) were required to practice the novel and control sequences daily for 3 months, using a home computer. Visual presentation and timing of the sequences was identical to that presented during the first scanning session (see Section “Study 1 – Fast Learning Phase”). Participants were requested to execute 22 repetitions of the novel sequence and 6 repetitions of the control sequence each day. They were also instructed to write down any incidence or missed practice and were contacted every week. A new scanning session was repeated at month 3 to evaluate brain activity during the SL phase. The experimental task performed during this scanning session was identical as that described for Study 1 (see Section “Study 1 – Fast Learning Phase”).

Recruitment criteria for this subgroup of subjects was as follows: Once participants underwent the first fMRI session of Study 1, they were randomly assigned into two different groups: (1) one group of participants (*n* = 12) who followed the experimental research explained in Study 2 (daily motor practice and fMRI scan after 3 months) and (2) one group of participants (*n* = 13) who underwent the same experimental protocol of Study 2, but who also received repetitive transcranial magnetic stimulation in the premotor cortex weekly. 2 participants (1 of group 1 and 1 of group 2) dropped out of the study due to different reasons: (1) lack of time to perform the daily motor practice and (2) not feeling secure about receiving transcranial magnetic stimulation. Only subjects who did not receive transcranial magnetic stimulation (*n* = 11) were included in this part of the study (Study 2).

### Behavioral Data

Behavioral data recorded during the scanning sessions were analyzed as follows: the average number of correct movements per sequence was calculated for every subject, task (novel and control) and scanning session (FL and SL) as a measure of motor performance. The maximum number of correct movements per motor sequence was 8. Additionally, the average duration of motor execution and the average inter-tap intervals per sequence were also calculated for every subject, task and scanning session to monitor motor accuracy. In this study, the control condition was used both as a measure of attentional control and as a comparative measure of performance during the different learning phases. Since motor performance variables were not normally distributed, non-parametric statistical analyses were carried out. Statistical analyses were performed using STATA 15.0 and R 3.6.1. Significance level was set at 0.05.

#### Study 1 – Fast Learning Phase

Differences in motor performance (i.e., number of correct movements, duration and inter-tap intervals) between novel and control tasks were assessed using Wilcoxon signed-rank test. In order to evaluate whether age was a significant predictor of motor performance, three linear regression models were estimated, one for each behavioral variable (dependent variable: behavioral data of the novel sequence, independent variable: age).

#### Study 2 – Fast and Slow Learning Phases

Data from Study 2 allowed to assess motor performance in SL and compare motor accuracy between learning phases. Differences in motor performance between learning phases were evaluated using non-parametric two-way repeated-measures Analysis of Variance (ANOVAs) (Noguchi et al., 2012) with factors task (novel and control) and learning phase (fast and slow) for number of correct movements, duration and inter-tap interval, respectively. *Post hoc* comparisons were assessed using Wilcoxon signed-rank tests and Bonferroni correction. In order to evaluate whether age was a significant predictor of motor performance, two different linear regression models were estimated using data from the novel sequence for each behavioral variable: (1) dependent variable: behavioral data of the FL phase, independent variable: age; (2) dependent variable: the difference in behavioral data between FL and SL phases, independent variable: age. In the first regression models (1), we expected to obtain the same results as in Study 1. In the second regression models (2), we aimed to assess whether age was a significant predictor of improvements in performance observed from FL to SL.

### Functional Magnetic Resonance Imaging

#### Functional Magnetic Resonance Imaging Data Acquisition

A 3.0 Tesla MR scanner (Siemens TRIO, Germany) and a 12-channel head coil were used to acquire the data. 295 volumes were obtained during the scanning session using a T2*-weighted gradient echo-planar imaging (EPI) sequence (repetition time/echo time [TR/TE] = 3000/30 ms, field of view [FOV] = 192 mm × 192 mm, flip-angle = 30°, 49 slices, resolution = 3 mm × 3 mm × 3 mm). The anatomical image was acquired using a T1- weighted MPRAGE sequence [TR/TE = 1620/3 ms, inversion time (TI) = 950 ms, FOV = 250 mm × 187 mm × 160 mm, flip-angle = 15°, 160 slices, resolution = 1 mm × 1 mm × 1 mm].

#### Functional Magnetic Resonance Imaging Data Analyses

##### Pre-processing of Functional Magnetic Resonance Imaging Data

Functional data were realigned to the first volume of the series using the realign and unwarp procedure (Andersson et al., 2001). Field map data was not measured so it was not included in these analyses. In the case of data from Study 2, functional images were realigned to the first volume obtained in Study 1. Functional images were then co-registered to the anatomical image. A slice-timing correction and outlier detection procedure were also applied. Functional and anatomical images were then normalized into Montreal Neurological Institute (MNI) standard space and segmented into gray matter, white matter and cerebrospinal fluid (CSF) regions. Finally, a three-dimensional Gaussian smoothing kernel of 8 mm full width at half maximum was applied to the functional images.

##### Task-Evoked Activity Analyses

All analyses were carried out using SPM12 (Wellcome Department of Cognitive Neurology, London, United Kingdom) and were corrected using Family Wise Error (FWE) cluster level corrected procedures (*p* < 0.05 at cluster level -voxel defining threshold *p* = 0.001). Cerebral and cerebellar activation clusters were localized using the Statistical parametric mapping (SPM) Anatomy toolbox.

In this study, brain activity in the novel task during FL and SL was compared with rest, allowing us to assess altogether visuospatial processing, movement preparation and motor performance throughout the course of learning.

###### Study 1 – Fast Learning Phase

Tasks (visual novel, motor novel, visual control, motor control, and rest) were included in first level analyses for every subject. Individual data were modeled with a hemodynamic response function (Canonical HRF). Onset times for each event corresponded to: (1) visual novel and visual control tasks: the beginning of the visual presentation of the motor sequence (in this part of the paradigm, participants had to look at the targets of movement, but not to perform the motor sequence); (2) motor novel and motor control tasks: the presentation of the fixation cross, not discriminating between key presses (in this part of the task, participants were required to execute the visually presented motor sequence), (3) rest: the presentation of the fixation cross. Duration time of these events was set to: (1) visual novel and visual control tasks: 11.9 s (total duration of the visual presentation); (2) motor novel and motor control tasks: the total number of seconds that took each participant to execute the 8 finger motor sequence; (3) rest task: 4 s. Six motion regressors were included to control for movement generated during image acquisition. The motor novel condition was used in order to assess motor learning changes in activity and connectivity. From now on, when we refer to the Novel task, we will be using the motor novel task. A one-sample *t*-test using first level contrasts Novel > Rest was subsequently carried out at the group level in order to determine brain regions activated during FL. Additionally, in a separate analysis, age was included as a covariate in the one-sample t-test in order to assess age-related activity changes during FL.

###### Study 2 – Fast and Slow Learning Phases

Data from Study 2 allowed to assess brain activity during SL and changes in activity between learning phases. In the firs-level analysis, tasks (visual novel, motor novel, visual control, motor control, and rest) were included for every subject from Study 2. Individual data were modeled with a hemodynamic response function (Canonical HRF). Onset times for each event corresponded to: (1) visual novel and visual control tasks: the beginning of the visual presentation of the motor sequence (in this part of the paradigm, participants had to look at the targets of movement, but not to perform the motor sequence); (2) motor novel and motor control tasks: the presentation of the fixation cross, not discriminating between key presses (in this part of the task, participants were required to execute the visually presented motor sequence), (3) rest: the presentation of the fixation cross. Duration time of these events was set to: (1) visual novel and visual control tasks: 11.9 s (total duration of the visual presentation); (2) motor novel and motor control tasks: the total duration of the participant’s execution of the 8 finger motor sequence; (3) rest task: 4 s. Six motion regressors were included to control for movement generated during image acquisition. The motor novel condition was used in order to assess motor learning changes in activity and connectivity. From now on, when we refer to the Novel task, we will be using the motor novel task. Data for each of the corresponding learning phases (FL, SL) were modeled separately. A one-sample *t*-test using first level contrast Novel_SL > Rest_SL was subsequently carried out in order to determine brain regions activated during SL. One paired *t*-test was carried out in order to assess significant differences in activity between FL and SL phases. Additionally, in a separate analysis, age was included as a covariate in the one-sample *t*-test in order to assess age-related changes in activity during SL.

##### Functional Connectivity Analyses

All analyses (using the pre-processed fMRI data) were performed using the CONN toolbox v19c^1^, RRID:SCR_009550).

Connectivity analyses on pre-processed images were performed based on the following steps: (1) noise correction procedures which included noise components from white matter and CSF regions, subject-motion parameters, outliers and scrubbing, and constant session and task effects, (2) filtering for low-frequency signals (0.008-Inf Hz), and (3) generalized Psycho-Physiological Interactions (gPPI) seed-to-voxel analyses. gPPI analyses allowed to measure changes in functional connectivity which covaried with experimental factors.

Two sets of regions of interest (ROIs) were defined as seeds for connectivity analyses using data from Study 2: (1) in order to evaluate functional connectivity of main motor learning hubs, the first set of ROIs consisted of regions that showed significant activity both in FL and SL phases (i.e., significant clusters resulting from the conjunction analysis of the Novel_SL > Rest_SL and Novel_FL > Rest_FL contrasts); (2) in order to study functional connectivity of brain regions uniquely activated during FL or SL, the second set of ROIs was based on regions which showed significant differences in activity between FL and SL phases (i.e., significant clusters resulting from the second-level paired t-test analysis). All ROIs were defined as 5-mm spheres centered on the MNI coordinates of peak voxel activity in each cluster. A total of 9 ROIs (see Figure 2 and Tables 1, 2) were identified in these analyses.

**TABLE 1:** Cerebral regions showing significant activity in both FL and SL (*p* < 0.05, FWE corrected at cluster-level -voxel defining threshold *p* = 0.001).

Cluster size	Anatomical area	MNI coordinates	*T*-value
5202	**Postcentral gyrus**	**(L) −36 −28 50**	**10.21**
	Precentral gyrus	(L) −42 −24 64	9.38
	Inferior parietal lobule	(L) −48 −26 38	7.73
	SMA	(L) −6 −6 54	7.27
1441	**Cerebellum lobule VI**	**(R) 20 −50 −22**	**10.48**
	Cerebellar vermis lobule V	(R) 4 −58 −12	8.07
504	**Putamen**	**(L) −26 −6 2**	**6.67**
	Thalamus	(L) −16 −18 6	5.11
479	**Cerebellum lobule VIIIa**	**(R) 22 −60 −52**	**10.42**
230	**Precentral gyrus**	**(L) −56 6 30**	**7.90**

*Seed regions employed for connectivity analyses are displayed in bold.*

**TABLE 2:** Cerebral regions showing significant increases in activity in FL with respect to SL (*p* < 0.05, FWE corrected at cluster-level -voxel defining threshold *p* < 0.001).

Cluster size	Anatomical area	MNI coordinates	*T*-value
2344	**Precuneus**	**(L) −12 −70 50** (R) 14 −70 48	**8.37** 7.28
	Superior parietal lobule	(L) −14 −66 44 (R) 28 −58 66	7.46 6.46
	Superior occipital gyrus	(R) 26 −66 44	6.62
	Middle occipital gyrus	(L) −26 −68 36 (R) 38 −72 34	6.60 6.57
	Inferior parietal lobule	(L) −30 −54 52	6.10
350	**preSMA**	**(R) 2 8 64** (L) −4 2 62	**6.91** 6.13
288	**Superior frontal gyrus**	**(L) −28 0 70**	**6.73**
183	**Precentral gyrus**	**(R) 54 10 40**	**7.16**
	Middle frontal gyrus	(R) 46 22 34	4.72

*No significant increases in activity were found in SL when compared to FL. Seed regions employed for connectivity analyses are displayed in bold.*

Based on these ROIs, a series of gPPI seed-to-voxel analyses were conducted. This method allowed us to explore task-modulated functional connectivity between the selected seed region (gPPI analyses were carried out for each of the 9 previously predefined ROIs) and every other voxel in the brain. All analyses were corrected using FWE-cluster level correction procedures (*p* < 0.05 at cluster -voxel defining threshold *p* = 0.001). In addition, corrected *p*-values from the gPPI analyses were further adjusted for analyses of multiple seeds using Bonferroni correction. As a result, only clusters that showed FWE cluster corrected *p*-values < 0.0055 (i.e., 0.05/9) in the gPPI analyses were considered significant. Cerebral and cerebellar clusters were localized using the SPM Anatomy toolbox.

###### Study 1 – Fast Learning Phase

Functional connectivity analyses were carried out to assess connectivity patterns during FL. Regions which showed significant increases in connectivity in the novel versus the rest task were identified.

In addition, age was included as a second-level covariate in order to evaluate age-related connectivity changes in the contrasts of interest (Novel > Rest).

###### Study 2 – Fast and Slow Learning Phases

Using data from Study 2, functional connectivity analyses had a twofold objective: (1) to assess connectivity patterns during SL. In this case, regions that showed significant increases in connectivity in the Novel_SL versus the rest task were identified, and (2) to evaluate changes in connectivity between FL and SL phases. In this case, areas that showed significant changes in connectivity between Novel_FL and Novel_SL conditions were identified.

In addition, age was introduced as a second-level covariate in these analyses in order to evaluate age-related connectivity changes in the contrasts of interest (Novel_SL > Rest and Novel_FL > Novel_SL, respectively).

## Results

### Behavioral Data Results

#### Study 1 – Fast Learning Phase

All subjects completed the novel sequence correctly at least once (the average number of correct movements performed by the subjects along the 16 repetitions of the novel sequence can be found in Supplementary Figure 1).

**FIGURE 1:**
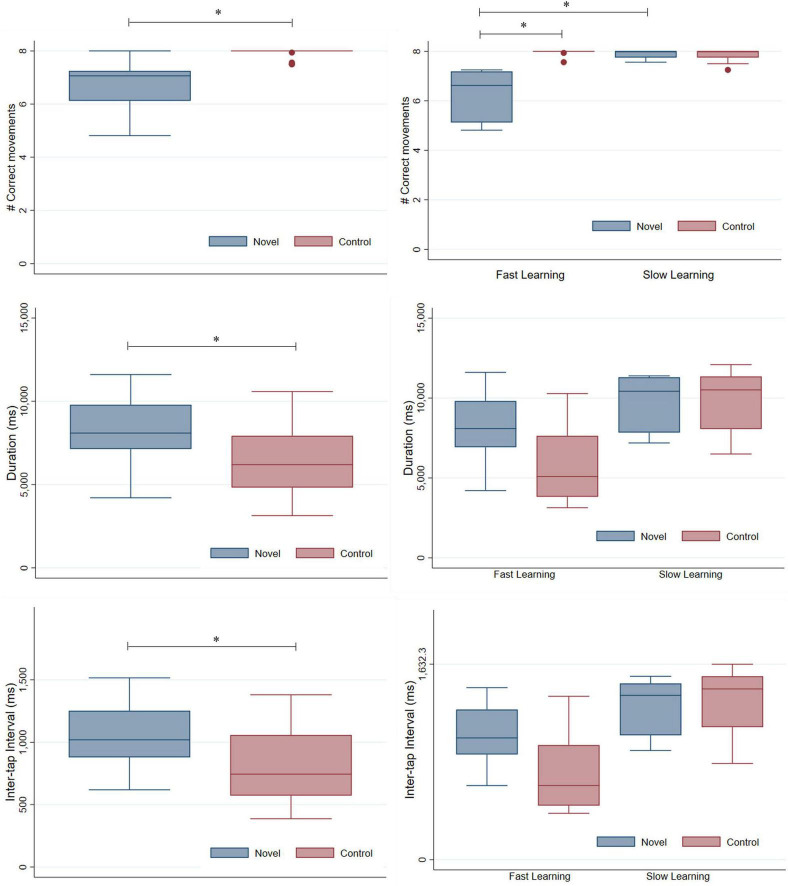
Box-plots for accuracy (i.e., average number of correct movements per sequence along the scanning session), duration and inter-tap intervals. The maximum number of correct movements which could be executed per sequence was 8, the maximum duration for motor execution was 12,500 ms and interstimulus interval during visual presentation was of 1,500 ms. **(Left)** Number of correct movements **(Top row)**, duration **(Middle row)**, and inter-tap interval **(Bottom row)** during FL in sample from Study 1 (*n* = 25). Different boxes are represented for novel and control sequences. **(Right)** Number of correct movements **(Top row)**, duration **(Middle row)** and inter-tap interval **(Bottom row)** during FL and SL in sample from Study 2 (*n* = 11). Different boxes are represented for every task (novel and control) and learning phase (FL and SL). * Indicates significant differences with *p*-values < 0.01 in every case.

As expected, the median number of correct movements differed significantly between novel and control sequences [*Median* (*Me*)*_*NOVEL*_* = 7.1, *Interquartile Range* (*IQR*)*_*NOVEL*_* = 6.1–7.3, *Me*(*IQR*)*_*CONTROL*_* = 8 (7.5–8); *z* = −4.280, *p* < 0.001, Wilcoxon signed-rank test]. Duration and inter-tap intervals also differed significantly between novel and control sequences, being greater in the novel sequence [Duration: *Me*(*IQR*)*_*NOVEL*_* = 8092.7 ms (7140.6–9792.7), *Me*(*IQR*)*_*CONTROL*_* = 6196.6 ms (4821.8–7925.7), *z* = 4.023, *p* < 0.001, Wilcoxon signed-rank test; Inter-tap interval: *Me*(*IQR*)*_*NOVEL*_* = 1019.5 ms (879.3–1252.3), *Me*(*IQR*)*_*CONTROL*_* = 743.4 ms (571.5–1058.4), *z* = 3.969, *p* < 0.001, Wilcoxon signed-rank test] (see Figure 1, left).

Age was not a significant predictor of any of the behavioral variables [linear regression, Correct movements: *F*(1,23) = 0.92, *p* = 0.3479; Duration: *F*(1,23) = 0.34, *p* = 0.5657; Inter-tap interval: *F*(1,23) = 0.04, *p* = 0.8348].

#### Study 2 – Fast and Slow Learning Phases

After the first fMRI scan, a subset of 11 subjects practiced daily the novel sequence for a period of 3 months. Participants reported high completion rates (i.e., percentage of days that completed training) of daily motor practice during this period (mean: 91.5%, ranging from 69 to 100%).

The median number of correct movements, duration and inter-tap intervals of sample from study 2 during FL and SL scanning sessions is displayed in the right side of Figure 1 (the average number of correct movements performed by the subjects along the 16 repetitions of the novel sequence can be found in Supplementary Figure 1).

Regarding the number of correct movements, significant interaction between factors Learning phase (FL and SL) and Task (novel and control) was found (Interaction: *ANOVA type statistic* = 52.29, *p* < 0.001; Learning phase: *ANOVA type statistic* = 15.80, *p* < 0.001; Task: *ANOVA type statistic* = 35.72, *p* < 0.001; non-parametric repeated measures ANOVA). *Post hoc* analyses revealed: (1) significant differences in the number of correct movements between novel and control tasks in FL [*Me*(*IQR*)*_*NOVEL*_* = 6.3 (5.1–7.2), *Me*(*IQR*)*_*CONTROL*_* = 8 (8–8), Wilcoxon signed-rank test, *z* = −2.936, *p* = 0.0033]; (2) non-significant differences in the number of correct movements between novel and control tasks in SL [*Me*(*IQR*)*_*NOVEL*_* = 8 (7.8–8), *Me*(*IQR*)*_*CONTROL*_* = 8 (7.8–8), Wilcoxon signed-rank test, *z* = 0.660, *p* = 0.5095]; (3) significant differences in the number of correct movements executed in FL and SL for the novel task (*z* = 2.936, *p* = 0.0033, Wilcoxon signed-rank test); (4) non-significant differences in the number of correct movements executed in FL and SL for the control task (*z* = −0.989, *p* = 0.3227, Wilcoxon signed-rank test). On the other hand, age was not a significant predictor of the number of correct movements executed in FL [*F*(1,9) = 0.95, *p* = 0.3545, linear regression] or of the changes in novel motor performance between FL and SL phases [*F*(1,9) = 0.32, *p* = 0.5865, linear regression].

Duration and inter-tap interval did not show significant interaction between factors Learning phase and Task (non-parametric repeated measures ANOVA: Duration: *ANOVA type statistic* = 0.22, *p* = 0.64; Inter-tap interval: *ANOVA type statistic* = 2.96, *p* = 0.086). Main effects of Learning phase and Task were close to the limit of significance for duration (Learning phase: *ANOVA type statistic* = 3.61, *p* = 0.057; Task: *ANOVA type statistic* = 2.82, *p* = 0.093, non-parametric repeated measures ANOVA). Inter-tap interval showed a non-significant effect of Learning phase and the main effect of Task was close to the limit of significance (Learning phase: *ANOVA type statistic* = 0.93, *p* = 0.34; Task: *ANOVA type statistic* = 3.26, *p* = 0.071, non-parametric repeated measures ANOVA). Finally, age was not a significant predictor of duration [*F*(1,9) = 0.88, *p* = 0.3732, linear regression] or inter-tap intervals in FL [*F*(1,9) = 0.34, *p* = 0.5719, linear regression] or of the changes in novel motor performance between FL and SL [Duration: *F*(1,9) = 0.15, *p* = 0.7085; Inter-tap interval: *F*(1,9) = 0.02, *p* = 0.8827, linear regression].

### Functional Imaging Results

This study aimed to assess brain activity and connectivity patterns over the course learning (FL and SL) and whether these patterns were correlated with age.

#### Task Evoked Activity Analyses

**FIGURE 2:**
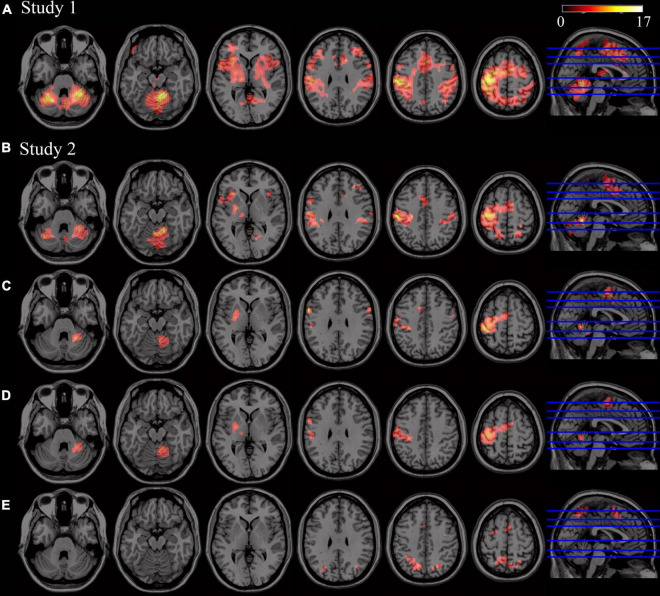
Patterns of activity related to motor learning (*p* < 0.05, FWE corrected at cluster-level -voxel defining threshold *p* = 0.001). **(A)** Significant activity during FL (second level analysis using Novel > Rest contrasts, using sample from Study 1, *n* = 25). **(B)** Significant activity during FL (second level analysis using Novel > Rest contrasts, using sample from Study 2, *n* = 11). **(C)** Significant activity during SL (second level analysis using Novel_SL > Rest contrasts). **(D)** Brain regions showing significant activity in both FL and SL phases (conjunction analysis of the Novel_FL > Rest_FL and Novel_SL > Rest_SL contrasts). **(E)** Brain regions showing significant increases in activity in FL with respect to SL phase (i.e., significant clusters resulting from second-level paired *t*-test analysis). No significant increases in activity were found in SL when compared to FL.

Brain regions activated during FL vs. rest are displayed in Figure 2A (using sample from study 1). Bilateral activation in cerebellum (lobules I–IV, V, VI, VII, and VIII), parietal, premotor, motor, dorsolateral cortices, pre-supplementary motor area/supplementary motor area (preSMA/SMA) regions and striatum was identified.

Brain regions activated during SL are displayed in Figure 2C (using sample from study 2). The right cerebellum (lobules V, VI, and VIII), left motor areas, left SMA and the left putamen showed significant activity during SL when compared to rest. Regions activated during FL vs. rest in this subsample are also presented in Figure 2B. As expected, due to the smaller sample size, brain regions involved in FL were similar to those obtained in study 1, but to a lesser extent.

Brain regions that showed significant activity both in FL and SL phases are displayed in Figure 2D and Table 1 respectively. The right cerebellum (VI and VIII), left posterior putamen, left precentral and postcentral gyrus and SMA were mainly recruited throughout the course of learning (FL and SL). On the other hand, regions that showed significantly increased activity in FL versus SL were mainly located in the precuneus, preSMA, left superior frontal gyrus and right precentral gyrus (see Table 2 and Figure 2E). No significant increases in activity were found in SL when compared to FL.

The inclusion of age as a covariate in the above analyses did not show any significant associations between activity and age.

#### Functional Connectivity Analyses

**FIGURE 3:**
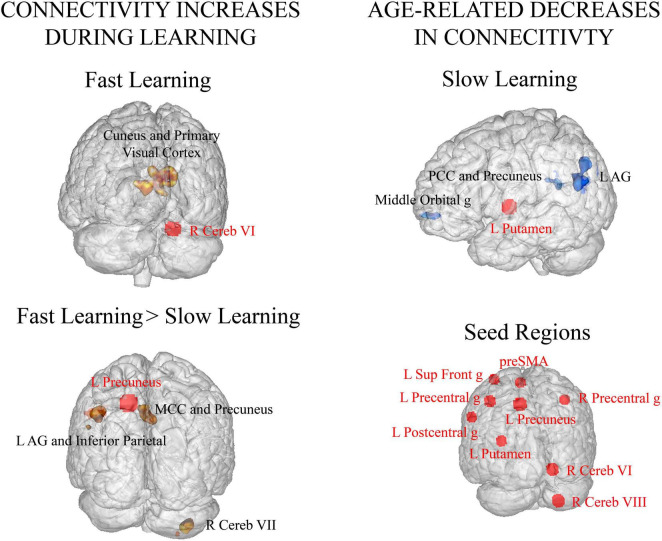
Seed-to-voxel functional connectivity analyses [*p* < 0.0055 (after Bonferroni correction for multiple seeds), FWE corrected at cluster-level -voxel defining threshold *p* = 0.001]. Seed regions are displayed with red circles and red font and their targets with black font. **(Left column)** Functional connectivity increases related to motor learning. **(Top row)** Significant increases in connectivity during FL compared to rest. **(Bottom row)** Significant increases in connectivity in FL with respect to SL. **(Right column) (Top row)** Age related decreases in functional connectivity during SL vs. rest. **(Bottom row)** Seed regions included in the connectivity analyses. R: right, L: left, AG: angular gyrus, MCC: middle cingulate cortex, PCC: posterior cingulate cortex, g: gyrus.

Significant increments in connectivity occurred mainly during FL. Specifically, right cerebellum (lobule VI) showed greater connectivity with high-order and visual processing areas (cuneus, calcarine gyrus, primary visual cortex and lingual gyrus) in FL versus rest (see Table 3 Fast Learning and Figure 3 Fast Learning). In addition, the left precuneus showed greater connectivity within other parietal regions (precuneus, angular gyrus, and inferior parietal lobule), with middle cingulate cortex, right cerebellum (lobule VIIa Crus I and VIIa Crus II) and with the left middle occipital gyrus in FL versus SL phase (see Table 3 Fast Learning > Slow Learning and Figure 3 Fast Learning > Slow Learning). No significant increments in connectivity were found in the SL phase compared to rest.

**TABLE 3:** Connectivity increases related to motor learning [*p* < 0.0055 (after Bonferroni correction), FWE corrected at cluster-level -voxel defining threshold *p* = 0.001].

Fast learning			
**Seed region: cerebellum lobule VI (MNI coordinates: 20 −50 −22)**			
**Cluster size**	**Anatomical area**	**MNI coordinates**	***F*-value**
320	Cuneus	(R) 6 −68 20 (L) −6 −74 20	22.38 19.36
	Calcarine gyrus	(R) 14 −70 18 (L) −6 −60 6	20.45 21.46
	Primary visual cortex	(R) 4 −56 8	19.27
	Lingual gyrus	(R) 12 −54 4	17.17

**Fast learning > slow learning**			
**Seed region: left precuneus (MNI coordinates −12 −70 50)**			
**Cluster size**	**Anatomical area**	**MNI coordinates**	***F*-value**
297	Precuneus	(L) −2 −48 36	75.03
	Middle cingulate cortex	(R) 2 −46 34 (L) −4 −38 36	59.39 43.75
229	Cerebellum lobule VIIa Crus II	(R) 30 −70 −52	102.8
	Cerebellum lobule VIIa Crus I	(R) 12 −82 −28	30.53
215	Angular gyrus	(L) −46 −58 28	56.88
	Inferior parietal lobule	(L) −34 −74 44	55.08
	Middle occipital gyrus	(L) −34 −68 36	32.28

*gPPI analyses were carried out for every of the 9 ROIs. Only ROIs that showed significant task-modulated functional connectivity with other brain regions are displayed.*

On the other hand, significant decreases in connectivity during SL when compared to rest were found with aging. Specifically, the left putamen showed lower connectivity with the parietal cortex (angular gyrus and precuneus), the posterior cingulate and the middle orbital gyrus as age of the participants was greater (see Table 4 Slow Learning and Figure 3 Age related decreases in connectivity – Slow Learning).

**TABLE 4:** Age related significant decreases in connectivity [*p* < 0.0055 (after Bonferroni correction), FWE corrected at cluster-level -voxel defining threshold *p* = 0.001].

Slow learning			
**Seed region: left putamen, MNI coordinates −26 −6 2**			
**Cluster size**	**Anatomical area**	**MNI coordinates**	***F*-value**
343	Angular gyrus	(L) −52 −60 24	130.48
134	Precuneus	(R) 6 −46 20 (L) −2 −58 14	91.97 59.07
	Posterior cingulate cortex	(L) −4 −52 28	36.85
100	Middle orbital gyrus	(R) 4 54 −10 (L) 0 64 −8	76.35 56.93

*gPPI analyses were carried out for every of the 9 ROIs. Only ROIs that showed significant age-related connectivity changes in the contrasts of interest are displayed.*

## Discussion

In relation to fast motor learning (FL), all subjects learned the motor sequence and exhibited rapid improvements in motor accuracy. Motor performance during this learning phase showed strong bilateral activity among sensorimotor, dorsal premotor, parietal and dorsolateral prefrontal cortices, striatum, cerebellum (lobules I-IV, V, VI, VII, and VIII) and preSMA/SMA, in accordance with previous motor learning literature (Hikosaka et al., 2002; Dayan and Cohen, 2011; Doyon et al., 2018). Functional connectivity analyses also revealed a central role of cerebellar sensorimotor and associative regions during FL. On one hand, greater connectivity between lobule VI of the cerebellum and primary and high order visual processing areas was found in FL. It is important to note that motor learning was assessed using the period of time where subjects prepared and executed the sequential movement, right after the visual presentation of the novel motor sequence. In line with previous studies, the significant increase in connectivity found in FL between the cerebellum lobule VI and the cuneus extended to visual cortex, may be the result of the acquisition of an optimal internal model for sequence performance and to control for the on-going movement (Imamizu et al., 2000), or it may be the result of visual imagery of motion, evoking the novel motor sequence order and timing as it was displayed visually to participants (Lee et al., 2012). On the other hand, compared to SL, FL also showed significant increments in connectivity between precuneus and areas associated with attentional control and visuo-spatial processing: posterior cerebellum lobule VII -Crus I and II-, angular gyrus, inferior parietal, middle occipital and middle cingulate cortex regions. Therefore, our results suggest that strengthening these connections was required for the development of new visuospatial motor representations. The posterior region of the precuneus has been strongly related to successful memory retrieval, specially of spatial representations of sequential movements with reference to memorized patters. Also, the posteromedial parietal cortex and lateral parietal areas have a central role in elaborating information about egocentric and allocentric spatial relations for body movement control (Cavanna and Trimble, 2006). Both successful spatial memory retrieval and adequate proprioception of movement control would be essential for the correct execution of the novel motor sequence during FL. Our findings are in agreement and support the parietal memory network (PMN) termed by Gilmore et al. (2015) in their review. Gilmore et al. (2015) found that meta-analyses of memory encoding and retrieval consistently involved the precuneus, the mid-cingulate cortex and the posterior inferior parietal lobule/dorsal angular gyrus. In addition, resting state functional connectivity analyses evidenced that these three regions constituted a functional network that was different from the neighboring default mode network. Also in accordance with our results, Zhang and Li (2012) demonstrated that the ventral precuneus had greater connectivity with occipital and posterior parietal cortices. The precuneus also shows functional connectivity with multiple cerebellar areas, such as lobule VII, Crus I and Crus II (Zhang and Li, 2012). Regarding cerebellar regions, it has been proven that they play a major role in the detection and correction of errors due to visuomotor transformation (Halsband and Lange, 2006), which was crucial during FL. In addition, since participants were required to reproduce the motor sequence at the same frequency as it was presented, previous work suggests that parietal areas would be mainly involved in learning the finger sequence order whereas cerebellar regions would be in charge of learning the timing of the sequence (Sakai et al., 2002).

In contrast with the model proposed by Doyon et al. (2018), we did not find hippocampal activity during FL. One plausible explanation for our results may be the inclusion of the whole scanning session in the FL analyses. Previous work, using similar experimental tasks, showed hippocampal activation during later stages of FL (i.e., when subjects perform the task with few or no errors) approaching consolidation phase (Jenkins et al., 1994; Fernandez-Seara et al., 2009; Gheysen et al., 2010; Lungu et al., 2014; Eudave et al., 2017; Burman, 2019; Jacobacci et al., 2020). On the other hand, as previously stated, most of previous learning studies have been performed by young subjects and therefore the lack of hippocampal activity found in our work might also be the result of normal aging. Supporting this hypothesis, there is growing evidence that the hippocampus presents structural (Li et al., 2018; Li and King, 2019) and functional changes with aging that affect learning (Rapp, 1998; Sullivan et al., 2005; Ta et al., 2012; Nordin et al., 2017; Li and King, 2019; Diersch et al., 2021). Specifically, deficits in establishing spatial representations of novel environments that experience older adults might be due to larger excitability of the anterior hippocampus (Diersch et al., 2021). Also, declines in hippocampal functioning with aging have been associated with deficits in learning and memory (Rapp, 1998; Nordin et al., 2017) and significant reduction of anterior hippocampal activity with aging has also been related to changes in the volume or structural atrophy of this region (Ta et al., 2012; Nordin et al., 2017). Future research should further explore age-related changes in hippocampal activity during motor sequence learning.

During SL, subjects performed accurately the task and, as expected, employed less cerebral resources to perform the same motor sequence (Berlot et al., 2020). Most of the cortical activity in parietal, prefrontal cortices and preSMA disappeared and activity in the sensorimotor cortex, SMA, putamen and cerebellum (lobules V, VI, and VIII) became unilateral. Sensorimotor cortex, SMA and putamen have been long recognized as key regions in SL (Dayan and Cohen, 2011). However, the role of the cerebellum in SL is still under debate. Some authors argue that the cerebellum plays an important role in the precise representation of the temporal information of a sequential movement (Sakai et al., 2002; Wadden et al., 2013), as well as in sensorimotor (Salman, 2002; Yihong et al., 2005) and supervised learning (Doya, 2000). Others recognize that greater activation of the cerebellum in SL might be indicative of the formation of internal models for optimal sequence execution and for error correction (Penhune and Steele, 2012; Wymbs and Grafton, 2015). On the contrary, there are authors that consider a specific role of the cerebellum only in motor adaptation and not in SL (Doyon et al., 2003; Lehericy et al., 2005). Our results show a prevalent role of the hand sensorimotor cerebellum, lobules VI and VIII of posterior cerebellum, and of the lobule VII -Crus I and Crus II- in the posterior cerebellum throughout the course of learning. Specifically, the cerebellum showed significant activity in both learning phases in lobules VI and VIII of the sensorimotor cerebellum. Cerebellar regions also showed significant increments in connectivity during FL: between the cerebellar lobule VI and the cuneus and primary visual cortex and between the precuneus and the cerebellar lobule VII – Crust I and II-. In line with our results, there is growing evidence that supports a central role of the posterior cerebellum during learning for motor, cognitive and perceptual functions, whereas anterior regions would be mostly related with motor control (Salmi et al., 2010; Stoodley et al., 2012; Deluca et al., 2014; Guell and Schmahmann, 2020). Due to existent controversy on the role of the cerebellum in SL and previous research indicating that older adults demonstrate deficits in motor sequence memory consolidation (King et al., 2013), further research should assess whether the involvement of cerebellar regions in SL found in the present work might be the result of compensatory mechanisms that appear in late middle adulthood (i.e., due to the need of increased supervised learning and/or the recruitment of additional brain resources to perform accurate movements).

Regarding the effect of aging in motor learning during late middle adulthood, we did not observe changes in either motor performance or in brain activity during SL or FL. However, aging affected functional connectivity patterns of SL. Specifically, a reduction in connectivity between the contralateral posterior putamen and the angular gyrus, the precuneus, the posterior cingulate cortex and the middle orbital gyrus was found as the age of the participants was greater. Greater activity in the anterior striatum has been related to FL in previous work, whereas posterior putamen is mainly involved in SL/automatization (Steele and Penhune, 2010). In this line, there is extensive evidence that indicates that the striatum is involved in motor memory consolidation (Peigneux et al., 2003; Albouy et al., 2008) and in the storage of learned sequences (Floyer-Lea and Matthews, 2005; Doyon et al., 2009). In addition, the dorsolateral striatum is strongly connected to sensorimotor and parietal cortices and it has been demonstrated that this circuit has a major role in encoding motor associations (Penhune and Steele, 2012). The fact that aging produced a decrease in connectivity between the posterior putamen and the parietal memory network might be indicative of losses in network efficiency when approaching late adulthood. Also, the decrease in connectivity with age found between the posterior putamen and the medial prefrontal cortex is not surprising, since the frontal lobe is one of the main vulnerable regions in aging (Ren et al., 2013). Lastly, it is important to note that a dysfunction of the posterior putamen is mainly associated with difficulties in the automatic performance of learned motor skills, as it occurs in Parkinson’s disease. Our results might be indicative of the denervation of the posterior putamen with aging (de Keyser et al., 1990).

### Limitations

This study has a low sample size overall. In addition, from the initial study (Study 1, *n* = 25), the subset of subjects who participated in the longitudinal study (Study 2, *n* = 11) is even more limited. Altogether, the limited sample size of the study might have had an impact on statistical power. This study also lacks of a younger control group to assess which of the activity and connectivity patterns identified in the present study are specific to middle age. Moreover, long-term longitudinal data would have been needed to evaluate activity and connectivity changes with aging throughout middle adulthood. Lastly, the EPI sequence employed in the present study presented low temporal resolution (TR = 3 s). This resolution should be increased in future investigations.

## Conclusion

The present study provides a picture of performance, brain activity and functional connectivity patterns associated with motor sequence learning in late middle adulthood. As expected, our results confirmed that late middle-aged subjects improved their motor performance throughout the course of learning and that brain activity diminished from FL to SL. These findings were in accordance with brain activity patterns reported previously in studies of motor sequence learning in young subjects, except for the lack of hippocampal activity during FL and the involvement of cerebellum during SL. Future studies should confirm whether these differences are due to aging of the subjects or to other factors, such as experimental task or conditions.

To the author’s knowledge, this is the first study reporting functional connectivity patterns during motor sequence learning in late middle adulthood. Functional connectivity analyses were sensitive enough to detect changes in connectivity between learning phases and showed age-related connectivity changes, unlike analyses of brain activity. Our results showed that the precuneus and sensorimotor lobule VI of the cerebellum may play a central role in the establishment of new functional connections to create novel motor representations. In addition, we found a loss of functional network efficiency with aging between the posterior putamen and parietal and frontal regions when performing automatic movements. Our results might be indicative of the denervation of the posterior putamen with aging and may have important applications in motor rehabilitation programs.

## Data Availability Statement

The raw data supporting the conclusions of this article will be made available by the authors, without undue reservation.

## Ethics Statement

The studies involving human participants were reviewed and approved by Committee for Ethics in Research of the University of Navarra. The patients/participants provided their written informed consent to participate in this study.

## Author Contributions

MA-S, MF-S, and MP contributed to conception and design of the study. MA-S, MF-S, MP, FV, and FL collected the data. MA-S organized the data and performed all the analyses. MA-S, MF-S, MP, MM, EL, and LE interpreted the results. MA-S, MP, MM, EL, and LE wrote the first draft of the manuscript. All authors contributed to manuscript revision, read, and approved the submitted version.

## Conflict of Interest

The authors declare that the research was conducted in the absence of any commercial or financial relationships that could be construed as a potential conflict of interest.

## Publisher’s Note

All claims expressed in this article are solely those of the authors and do not necessarily represent those of their affiliated organizations, or those of the publisher, the editors and the reviewers. Any product that may be evaluated in this article, or claim that may be made by its manufacturer, is not guaranteed or endorsed by the publisher.

## Footnotes

1
www.nitrc.org/projects/conn
