# Supplementary material for: Brain Activity and Functional Connectivity Patterns Associated With Fast and Slow Motor Sequence Learning in Late Middle Adulthood
Source: Front Aging Neurosci. 2022 Jan 13;13:778201. doi: 10.3389/fnagi.2021.778201 (PMC8792532; doi:10.3389/fnagi.2021.778201)
Supplement: Supplementary file 1 [file Data_Sheet_1.PDF]

Supplementary Material

1 Supplementary Figures and Tables

1.1 Supplementary Tables

Table S1. Age and sex of participants included in the study (ordered by age). Subjects who carried out FL and SL tasks are highlighted in bold.

Participant	Age	Sex
1	42	M
2	45	F
**3**	**46**	**M**
4	46	M
5	47	F
**6**	**48**	**F**
7	48	F
8	50	F
**9**	**51**	**M**
10	53	M
**11**	**55**	**M**
**12**	**56**	**F**
13	57	M
**14**	**57**	**M**
**15**	**59**	**M**

16	59	M
17	59	F
18	61	F
**19**	**61**	**M**
20	63	M
**21**	**66**	**F**
**22**	**67**	**M**
**23**	**67**	**M**
**24**	**72**	**M**
25	72	M

1.2 Supplementary Figures

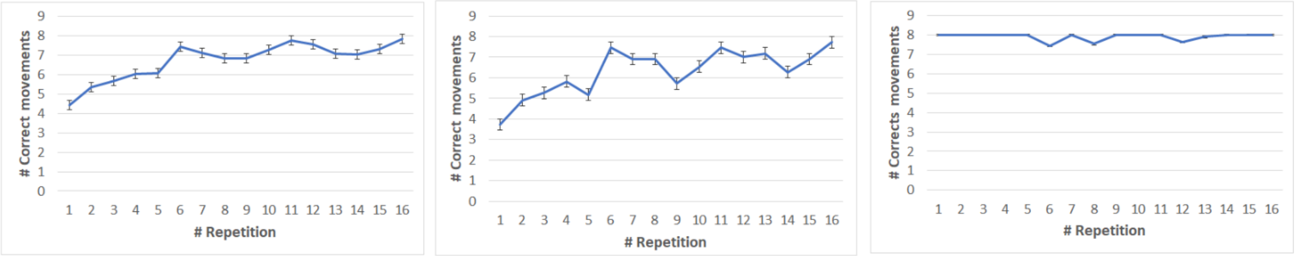

Fast Learning (n=25) Fast Learning (n=11) Slow Learning (n=11)

Supplementary Figure 1. Accuracy (i.e. average number of correct movements) across novel sequence repetitions. (A) Fast Learning performance in sample from Study 1. (B) Fast Learning performance in sample from Study 2. (C) Slow Learning performance related to sample from Study 2. Bars represent standard error.
